# The High Five Model as a Predictor of Optimal Functioning in University Students: A Longitudinal Study

**DOI:** 10.5964/ejop.15589

**Published:** 2026-05-29

**Authors:** Diana García Dilba, Alejandro Castro Solano

**Affiliations:** 1 Psychology Department, Universidad de Palermo, Buenos Aires, Argentina; 2 National Council for Scientific and Technical Research, CONICET, Buenos Aires, Argentina; 3 Psychology Department, Universidad de Palermo, Buenos Aires, Argentina; 4 Psychology Department, Universidad de Buenos Aires, Buenos Aires, Argentina; Stanford University, Stanford, CA, USA

**Keywords:** high factors, high five model, academic performance, psychopathological symptoms, psychological well-being

## Abstract

**Objectives:**

The high-five model (HFM) categorizes five positive human characteristics—erudition, peace, joviality, honesty, and tenacity — on the basis of an inductive psycholexical approach. This study aimed to evaluate the predictive power of the HFM in a sample of university students to assess whether these traits could predict optimal functioning after 20 months. Optimal functioning was defined as high academic performance; emotional, personal, and social well-being; and low levels of psychopathology.

**Methods:**

The study included 136 university students with a mean age of 30.8 years (*SD* = 1.86; 83% female; 17% male). The predictor variables were high factors (HFI – High Five Inventory –), and the criterion variables were emotional, personal, and social well-being (Mental Health Continuum-Short Form – MHC-SF –), psychopathological symptoms (Symptom Checklist-27, SCL-27), and academic achievement (self-reported academic grades). The data were analyzed both cross-sectionally and longitudinally.

**Results:**

The findings indicated that the HFI, particularly the factors of Honesty (β = .64, *p* = .05) and Tenacity (β = .49, *p* = .01), demonstrated high predictive power in identifying profiles of optimal functioning (high complete mental well-being) in university students after 20 months. The predictive power of the model was greater when predictors and criteria were analyzed cross-sectionally rather than longitudinally (explaining 35% vs. 17% of the variance).

**Conclusions:**

HFM effectively distinguished between high- and low-complete mental well-being groups both cross-sectionally and longitudinally, demonstrating its robustness in predicting optimal functioning at various time points.

The High-Five Model (HFM), proposed by Cosentino and Castro Solano (2017), focuses on positive personality traits and is rooted in positive psychology. Since its inception, the identification of human strengths and virtues — understood as positive aspects of individuals — has been a fundamental pillar of this psychological movement (Dahlsgaard et al., 2005). Over thousands of years, various religious and philosophical traditions, both in Eastern and Western cultures, have explored human virtues (Cosentino, 2010). However, it was not until the 20^th^ century that psychology addressed them scientifically. Perhaps one of the most well-known taxonomies for identifying human strengths and virtues was designed by Peterson and Seligman (2004), who proposed 24-character strengths. Over the past two decades, this model has been extensively studied and has had a broad influence on positive psychology (Ruch & Stahlmann, 2019).

The HFM is a model that considers positive traits from an inductive psycholexical approach. Its development was based on the everyday language people use to describe the positive characteristics they attribute both to themselves and others, going beyond moral attributes. To construct this model of socially shared positive traits, the authors designed the High Five Inventory (HFI), an instrument consisting of 23 items organized into five subscales (Cosentino & Castro Solano, 2017). The HFM employs statistical and syntactic criteria instead of semantic criteria to avoid theoretical biases and enable replicability in different populations (Castro Solano & Cosentino, 2019). The five positive factors identified by the HFM — erudition, peace, joviality, honesty, and tenacity — maintain a conceptual and empirical relationship with the factors of the Big Five Model (BFM), although they are not mere duplications. While the HFM factors are considered the positive poles of the BFM factors, they represent stable, measurable positive psychological characteristics that can vary among individuals and may increase or decrease due to internal or external influences (Castro Solano & Cosentino, 2019; Cosentino & Castro Solano, 2017; Quito Calle & Cosentino, 2024).

The term “high” for the factors in the HFM was chosen for two main reasons. First, these factors are related to individual characteristics that ordinary people positively value. Second, they represent the positive poles of the BFM factors. For example, the factor of Erudition is linked to Openness to Experience, Peace to Emotional Stability (opposite to Neuroticism), Joviality to Extraversion, Honesty to Agreeableness, and Tenacity to Conscientiousness (Cosentino & Castro Solano, 2017).

Given the model’s recent development, empirical studies on the HFM remain limited. However, the findings obtained to date have already revealed significant associations between HFM factors and various relevant variables. First, initial studies confirmed the replicability of the factorial structure of the HFI in an adult population, as well as the incremental validity of the HFM factors in predicting different types of psychological well-being, outperforming the BFM (Cosentino & Castro Solano, 2017). Another study revealed that HFM traits, such as peace and joviality, were not only positively associated with psychological well-being indicators (flourishing) but also with a lower risk of medical conditions and allowed for differentiation between individuals with and without maladaptive personality traits (Castro Solano & Cosentino, 2017). Another study analyzing different profiles of internet users revealed that high levels of honesty and tenacity were negatively associated with excessive social media use (Lupano Perugini & Castro Solano, 2019). In the academic context, high factors, particularly tenacity — the positive trait of volition — and erudition — the positive trait of knowledge — have been demonstrated to be positively associated with academic performance and adjustment to university life (Castro Solano & Cosentino, 2019). Another study conducted on a large sample of Ecuadorian students revealed that high factors not only predict academic performance but are also superior to classic predictors of academic success, such as academic motivation, anxiety, and procrastination (Quito Calle & Cosentino, 2024). In recent research, the predictive power of high factors was evaluated alongside a model of negative traits to predict psychological well-being via machine learning algorithms. The study demonstrated that the integrated model of positive and negative traits (particularly erudition, tenacity, and malignancy), which is based on a psycholexical approach, has a predictive power comparable to or even superior to that of the BFM in predicting both hedonic and eudaimonic well-being (Castro Solano et al., 2024).

## Optimal Functioning

The concept of optimal functioning has its roots in humanistic psychology (Rogers, 1963). Within this approach, particular emphasis is placed on the positive aspects of personality, which are linked to psychological well-being and the full development of individuals. Although differing in their approach, authors within positive psychology have revisited this notion, relating it to theories of psychological well-being, particularly within the eudaimonic tradition. This perspective views well-being as intrinsically linked to personal fulfillment, growth, and the search for meaning in life (Huta & Waterman, 2014). One of the concepts most closely associated with optimal functioning is *flourishing*, which has been developed by several authors (Diener et al., 2010; Huppert & So, 2013; Keyes, 2005; Seligman, 2011). Keyes (2005) defines flourishing as high levels of emotional well-being (positive affect and life satisfaction), psychological well-being (autonomy, personal growth, positive relationships, environmental mastery, self-acceptance, and life purpose), and social well-being (social acceptance, actualization, contribution, social coherence, and integration).

Flourishing can be situated within the continuum of positive mental health proposed by Keyes and Lopez (2002). One end of the continuum is flourishing, which represents optimal functioning, whereas the other end is languishing, characterized by poor mental health (low levels of well-being), the opposite of complete mental health. Keyes (2005) views flourishing as the optimal state of mental health.

Keyes (2007) also integrated the conceptualization of mental health with the classification of mental disorders according to the DSM model, developing the *two continua model* (flourishing/languishing and the presence/absence of mental disorders). According to the author, mental health and mental illness are distinct but related variables, and numerous empirical studies have supported this assertion (Galderisi et al., 2015; Gilmour, 2014; VanderWeele, 2017). Complete mental health includes high levels of emotional, psychological, and social well-being, as well as the absence of psychological disorders.

In this study, we adopt Keyes’ (2005) two continua model as an indicator of optimal functioning. Given the focus on university students, we include academic performance as an additional indicator.

## Normal and Positive Personality Traits Linked to Optimal Functioning

Given the limited empirical evidence on the HFM model as a predictor of well-being and psychological disorders, we reviewed empirical studies that examine personality traits and character strengths — variables closely related to the model — and their connection to well-being and psychological disorders. Personality has been widely recognized as a robust predictor of subjective well-being, particularly when it is assessed through the BFM. A recent meta-analysis revealed that neuroticism, extraversion, and conscientiousness are strongly associated with both hedonic and eudaimonic well-being (Anglim et al., 2020).

Similarly, character strengths such as curiosity, gratitude, hope, love, and zest have been shown to significantly correlate with life satisfaction across various adolescent and adult samples and in different cultural contexts (e.g., Park et al., 2004; Park et al., 2006). In general, character strengths are associated with a lower prevalence of psychological disorders and positive mental health (Azañedo et al., 2021; Kumar et al., 2020). Kaufman et al. (2019)’s more recent model revealed that the three Light Triad personality traits (e.g., humanism, Kantianism, and faith in humanity) positively correlate with life satisfaction and contribute to overall well-being (Kaufman et al., 2019; Stavraki et al., 2023).

On the other hand, the relationship between personality traits and academic performance is well documented, with conscientiousness emerging as a consistent and robust predictor of academic success (see Mammadov, 2022). In particular, the conscientiousness factor of the BFM has consistently emerged as one of the strongest predictors of academic performance, as shown in previous research (Mitrofana & Ion, 2013). With respect to openness to experience, the results are mixed, with studies documenting weak to moderate positive effects on academic performance.

For character strengths, perseverance stands out as one of the strengths most correlated with academic performance (Browning et al., 2018; Lounsbury et al., 2009). Moreover, other strengths, such as curiosity, self-regulation, and love of learning, have also shown significant relationships with academic success (Wagner & Ruch, 2023).

With respect to HFM, high levels of tenacity and erudition are positively associated with academic performance and adjustment to university life (Castro Solano & Cosentino, 2019). Furthermore, these factors not only predict academic performance but also have been shown to be superior predictors compared with other classic variables, such as motivation, anxiety, and procrastination (Quito Calle & Cosentino, 2024).

The HFM has proven effective in predicting various psychological variables of interest (e.g., academic performance and adjustment, psychological well-being), often surpassing the predictive power of traditional personality traits. However, most studies utilizing the HFM have been cross-sectional. To address this gap, a longitudinal study was designed to evaluate the predictive power of the HFM over an extended period of nearly two years (20 months). The goal of this research was to verify whether the HFM can longitudinally predict optimal functioning in a group of university students.

Thus, the following hypothesis is proposed:

High factors predict optimal functioning (high emotional, personal, and social well-being; high academic performance; and low psychopathological symptoms) after *20 months.*

## Method

### Participants

A convenience sample of 136 voluntary participants (113 women, 83%) was included in the study. The sample was incidental and consisted of psychology university students residing in the Autonomous City of Buenos Aires, Argentina. The sample included students who had completed at least three years of a five-year degree program. The mean age was 30.8 years (*SD* = 1.86). In terms of socioeconomic status, the majority of the participants (95.6%) identified as belonging to the middle class (upper-middle, middle, or lower-middle), with only 3.7% reporting a high standard of living and 0.7% reporting a low socioeconomic level. Regarding employment status, 39.7% were employees, 19.1% were self-employed, 2.9% were employers, 1.5% reported working without pay, 6.6% identified as heads of household, and 30.1% reported being unemployed or not currently working.

### Instruments

#### High Five Inventory (HFI)

The HFI (Cosentino & Castro Solano, 2017) measures the five high-level factors of the HFM: peace, honesty, tenacity, erudition, and joviality. It consists of 23 items answered on a scale ranging from 1 (Never) to 7 (Always). The HFI has demonstrated convergent, divergent, and incremental validity beyond the facets and factors of the Big Five Model. The HFI showed good model fit in both the development sample (e.g., CFI = .968) and the validation sample (e.g., CFI = .963). The alpha and omega reliability coefficients for each factor were > .80. For this sample, the Cronbach’s alpha coefficients [Omega coefficients in brackets] were, *Peace* = .85 [.86], *Honesty* = .81 [.82], *Tenacity* = .84 [.85], *Erudition* = .81 [.82], and *Joviality* = .87 [.88].

#### Mental Health Continuum-Short Form (MHC-SF)

This 14-item instrument (MHC-SF) (Keyes, 2005; Argentine adaptation, Lupano Perugini et al., 2017) assesses the degree of emotional well-being, personal well-being, and social well-being. The items inquire about the frequency with which certain emotions have been experienced, using a Likert scale ranging from 0 (Never) to 5 (Every day). For this sample, the Cronbach’s alpha coefficients [Omega coefficients in brackets] were as follows: *Emotional well-being*: .84 [.85]; *Social well-being*: .75 [.77]; and *Personal well-being*: .85 [.86].

#### Symptom Checklist — 27 (SCL-27)

The SCL-27 (Hardt & Gerbershagen, 2001; Góngora & Castro Solano, 2021) is a short version of the SCL-90-R (Derogatis, 1977). It is composed of 27 items rated on a 5-point Likert scale ranging from 0 (Not at all) to 4 (Extremely), assessing psychopathological unspecified symptoms experienced in the previous week. In this study, the GSI was used as an indicator of psychopathological symptoms. The alpha coefficient for this sample was .84, and the omega coefficient was .85.

#### Academic Achievement

In the first data collection, information on sociodemographic variables (gender and age) and participants' self-reported academic grades (average, range 0–10) were collected. During the second data collection, the participants were asked to provide their current academic grades again. Academic achievement was defined as the overall average grade obtained by students across the courses they had completed throughout their university studies. The students were asked to self-report the grades recorded in their transcripts, which they could easily access online. Academic performance scores remained stable over the time interval considered (r1/r2 = .86).

### Procedure

The measures were administered on two separate occasions. The first assessment, conducted at the end of 2019, included the HFI, the MHC-SF, and the SCL-27 and collected information on academic achievement. This period preceded the COVID-19 pandemic and lockdown. The participants were reassessed 20 months after lockdown restrictions had been lifted. During this second assessment, the MHC-SF and SCL-27 were administered again, and updated information on academic performance was requested. The primary inclusion criterion was that participants be of legal age, specifically 18 years old or older. Surveys were administered online via SurveyMonkey. The first page of the survey requested participants' consent, ensuring the anonymity of their data and their use solely for research purposes. After being informed about the study’s objectives, the participants provided their informed consent. They were informed that they could withdraw from the study at any time without needing to justify their decision and without any penalty. A small percentage did not complete the second part of the study (18 participants, 13% of the total). The research was conducted in accordance with international ethical guidelines (APA and NC3R) and the regulations of the National Council for Scientific and Technical Research (CONICET) for ethical conduct in social sciences and humanities (Resolution No. 2857, 2006) and received approval from the *Ad Hoc Committee for Responsible Research Conduct* of the Facultad de Psicología, Universidad de Buenos Aires (Resolution No. 2016/156), within the framework of the project funded by the University of Buenos Aires through the UBACyT Program (project code 20020150100037BA), titled *Assessment of positive traits: Their relationship with personality disorder traits and psychological well-being*.

### Data Analysis

First, two cluster analyses (K-means) were conducted to identify groups of students with varying levels of functioning regarding psychological well-being, psychopathological symptoms, and academic performance, both at 0 and 20 months later (Time 1). Second, two logistic regression analyses were performed, with HFM factors included as predictors and cluster membership (at Time 0 and Time 1) included as outcome variables. The choice of this analytical strategy — cluster analysis followed by logistic regression — was primarily driven by the exploratory nature of the study and by practical considerations related to sample size. Data preparation was performed via SPSS Version 25. The data were analyzed via Jamovi (Version 2.4.1). For the cluster analysis, the Nbclust package in R (Version 4.3.3, R Core Team, 2024) was used. All the data, materials, analysis and codes used in this study are publicly available in the Open Science Framework (OSF) repository at Castro-Solano and Garcia Dilba (2026).

## Results

### Clusters With Differential Functioning in Academic Performance, Clinical Symptoms, and Psychological Well-Being (Time 0)

A cluster analysis using the K-means method was conducted to identify groups with high and low optimal functioning on the basis of levels of hedonic and eudaimonic well-being, psychopathological symptoms, and academic performance. This exploratory analysis aimed to group participants in such a way that minimizes the within-group distance and maximizes the distance between the centroids of the groups. The purpose of this technique is to assign subjects with similar characteristics in the selected variables to the same group (Everitt et al., 2011). Standardized scores were used for all variables, and the analysis was conducted via a Euclidean distance matrix. The Nbclust package was applied to determine the optimal number of clusters. Most indices suggest selecting two groupings on the basis of separation and cohesion (e.g., Calinski–Harabasz index = 37.23). Statistically significant differences were found between all the variables included in the clusters (ANOVA, *p* < .001), except for academic achievement, which did not differ between the two groupings (*F* (1, 134) = .84, *p* = .360). The first cluster, representing 58% of the sample (*n* = 80), consisted of participants with high levels of hedonic and eudaimonic well-being and a low presence of psychopathological symptoms and was labeled “complete mental well-being” (flourishing). The second cluster, representing 42% of the sample (*n* = 56), included individuals with low levels of both hedonic and eudaimonic well-being and medium to high scores in clinical symptomatology and was labeled “Low Complete Mental Well-Being” (Languishing).

### Relationships Among Clusters, Sociodemographic Variables, and High Factors (Time 0)

In the clusters of complete mental well-being (CMWB), no significant differences were found by age (*t* (134) = 1.72, *p* = .088, *d* = .30) or gender (χ^2^ (1, *N* = 136) = 2.69, *p* = .101). Logistic regression was performed with high factors as predictor variables and cluster membership as the criterion variable (1 = Low CMWB/2 = High CMWB). The model was statistically significant (χ^2^ (5, *N* = 135) = 41.30, *p* < .0001), indicating that the five predictors collectively distinguish between individuals belonging to one cluster or the other. The explained variance was 35% (Nagelkerke *R*^2^). The model’s accuracy in identifying true positive cases was 76%. It correctly identified 62% of the low-complete mental well-being cases and 86% of the high-CMWB cases. The area under the receiver operating characteristic curve (AUROC), which represents the balance between sensitivity and the true negative rate, was .80. The predictors that were significant in the model were erudition (β = .53, *p* = .05) and tenacity (β = .60, *p* = .01). These values suggest that the model performs well in correctly identifying cases of ‘High Complete Mental Well-Being’ but performs poorly in identifying cases of Low Complete Mental Well-Being.

### Clusters of Profiles With Differential Functioning in Academic Performance, Clinical Symptoms, and Psychological Well-Being (Time 1)

A cluster analysis using the K-means method was conducted to identify groupings reflecting optimal functioning at Time 1, following the same methodology as at Time 0. The Nbclust package was used to determine the optimal number of clusters. Most indices suggest selecting two clusters on the basis of separation and cohesion (e.g., Calinski–Harabasz index = 39.28). Statistically significant differences were found between all the variables included in the clusters (ANOVA, *p* < .001), except for academic achievement, which did not differ between the two clusters (*F* (1, 134) = 1.94, *p* = .166). The first cluster, representing 56% of the sample (*n* = 76), was characterized by high levels of both hedonic and eudaimonic well-being and low levels of psychopathological symptoms and was labeled the CMWB. The second cluster, representing 44% of the sample (*n* = 59), included participants with low levels of both hedonic and eudaimonic well-being and moderate to high levels of clinical symptoms and was labeled “Low CMWB”.

### Relationships Among Clusters, Sociodemographic Variables, and High Factors (Time 1)

In the CMWB clusters, differences were observed by age (*t* (133) = 2.69, *p* = .008, *d* = .47), but no significant differences were found by sex (χ^2^ (1, *N* = 135) = .06, *p* = .796). The high CMWB cluster consisted of younger individuals. Logistic regression was conducted with high factors as predictors and cluster membership (1 = Low CMWB/2 = High CMWB) as the outcome variable. The model was statistically significant (χ^2^ (5, *N* = 135) = 17.57, *p* < .004), indicating that the five predictors collectively distinguish between individuals in the different clusters. The explained variance was 17% (Nagelkerke *R*^2^). The model's accuracy in identifying true positives was 69%, correctly classifying 45% of the cases with low complete mental well-being and 87% of the cases with high CMWB. The AUROC was .70. The significant predictors in the model were honesty (β = .64, *p* = .05) and tenacity (β = .49, *p* = .01). These results suggest that the model performs well in identifying cases of high CMWB but has lower performance in identifying cases of low CMWB. In summary, the high-five model (HFM) of positive traits provides a reasonably good prediction of which individuals will flourish over a period of nearly two years.

In addition to identifying clusters at each time point, we examined the stability of individual cluster membership over time. A transition matrix revealed that 68 participants remained in the High CMWB group across both assessments, while 48 remained in the Low CMWB group. However, 12 participants shifted from High to Low functioning, and 8 transitioned from Low to High functioning. A McNemar test showed a statistically significant imbalance in these transitions (χ^2^ = 1.0, *p* < .001), indicating a greater tendency toward decreased optimal functioning over time.

## Discussion

The results of this study provide strong evidence for the predictive power of the HFM in identifying differences in optimal functioning over time, specifically over a period of approximately two years. The HFM effectively distinguished between high- and low-complete mental well-being groups both cross-sectionally and longitudinally, demonstrating its robustness in predicting optimal functioning at various time points. Tenacity emerged as the most predictive factor at both time points. These findings reinforce the idea that certain positive personality traits, related to perseverance and the ability to set and achieve goals through sustained effort, play crucial roles in maintaining optimal functioning over time. Tenacity is closely linked with the conscientiousness factor of the Big Five Model (BFM). This model posits that human personality is composed of five basic dimensions: neuroticism, extraversion, agreeableness, openness to experience, and conscientiousness (Costa & McCrae, 1992; Davis & Panksepp, 2018). Among these traits, conscientiousness and extraversion are considered protective factors against the development of psychological disorders (Lyon et al., 2021). Some researchers argue that individuals with high neuroticism, low extraversion, and low conscientiousness are more vulnerable to developing psychological issues (Kotov et al., 2010; Wardenaar et al., 2014). Therefore, individuals with high conscientiousness scores are less likely to develop psychological disorders and experience greater well-being.

In their synthesis of existing research, Anglim et al. (2020) identified conscientiousness, along with neuroticism and extraversion, as one of the personality factors most related to both hedonic and eudaimonic well-being. In line with these findings, previous research on positive personality traits has revealed a strong association between “light” personality traits (Light Triad: Kantianism, humanism, and humanity) and life satisfaction (Kaufman et al., 2019; Stavraki et al., 2023). Conversely, research on the Dark Triad model has demonstrated that negative personality traits — such as antagonism, disinhibition, and Machiavellianism — generally oppose the positive traits of BFM, except for neuroticism, and are negatively associated with eudaimonic well-being (Blasco-Belled et al., 2024; Liu et al., 2021). Soto (2015) conducted a large longitudinal study with an Australian sample and reported that individuals who were initially extraverted, agreeable, conscientious, and emotionally stable increased their well-being over time.

Previous studies utilizing the HFM have yielded results that are consistent with those of this research. Tenacity has been identified as a significant predictor of various positive outcomes, including responsible social media use (Lupano Perugini & Castro Solano, 2019), academic performance and adjustment to university life (Castro Solano & Cosentino, 2019), flourishing levels (Castro Solano & Cosentino, 2017), and academic performance beyond traditional predictors (Quito Calle & Cosentino, 2024).

Erudition (the positive trait of knowledge) was a better predictor of optimal functioning at Time 0, whereas honesty (the positive trait of agreeableness) was a better predictor at Time 1. Erudition is the positive extension of the openness-to-experience factor and the positive extension of the agreeableness trait of the BFM. The evidence regarding these two traits and psychological well-being is mixed.

Malouff et al. (2005) reported that individuals with low levels of agreeableness are at greater risk of developing psychological disorders. In this context, our findings are consistent with theirs. However, Kotov et al. (2010) did not observe a consistent association between agreeableness, openness to experience, and psychological disorders. Therefore, the evidence regarding this finding is mixed and not very clear.

Our model did not capture differences in academic achievement, and in this respect, these results are inconsistent with previous studies that highlight tenacity and erudition as important predictors of this variable, even beyond classic predictors (e.g., academic motivation, Quito Calle & Cosentino, 2024). A possible explanation could be that the sample was relatively small and very homogeneous, as the study design prioritized the availability of participants to be assessed twice over a period longer than a year rather than a greater heterogeneity to capture other individual differences.

Importantly, the predictive power of the model was lower at Time 1 than at Time 0. While at Time 0, the model explained 35% of the variance, at Time 1, it explained only 17%. This may suggest that, over time, other contextual or personal factors not considered in this study may have influenced individuals’ well-being. Notably, the period analyzed in this research covered approximately two years, almost the entire duration of the pandemic. Although predictor variables were administered before the pandemic and criterion variables after most confinement restrictions had been lifted, well-being was likely affected by the specific circumstances of the pandemic. The scientific literature provides ample evidence of increased psychological disorders and decreased psychological well-being as a result of both the pandemic and the lockdown (Holm-Hadulla et al., 2021; Möhring et al., 2021; O’Connor et al., 2021). This aligns with the period considered in this study between the measurement of predictor variables and criterion variables. However, more recent longitudinal research with nationally representative samples (Zaninotto et al., 2025) has shown that although there was a significant decline in happiness, eudaimonic well-being, and life satisfaction during the pandemic — especially in later phases — positive psychological functioning showed a notable recovery in subsequent years. In fact, eudaimonic well-being and life satisfaction exceeded pre-pandemic levels by 2023. These findings support the idea that although both assessments in our study were conducted during periods without active COVID-19 restrictions, residual emotional and contextual effects may still have influenced participants’ well-being.

The HFM model demonstrates high sensitivity in identifying individuals characterized as high-functioning optimal individuals (high well-being and low symptomatology). At both time points, the model exhibited excellent sensitivity (true positive rate) for the high factors (Time 0 = .86; Time 1 = .87). However, the model's performance in detecting low optimal functioning cases was less robust, with specificity — defined as the ability to avoid incorrectly classifying negative cases as positive — being lower than expected (specificity: Time 0 = .62; Time 1 = .44; AUROC: Time 0 = .80; Time 1 = .70). This finding indicates that while high factors are strong predictors of positive psychological outcomes, they are less effective in identifying negative outcomes. This limitation may suggest that the HFM has a bias toward positive outcomes, showing a greater tendency to predict flourishing rather than languishing. This finding aligns with previous research, which suggests that positive variables are often more effective at predicting positive outcomes than negative variables are. Specifically, studies on dispositional personality factors, such as strengths and virtues, highlight their role as significant predictors of positive functioning (Azañedo et al., 2021; Peterson & Park, 2011; Proctor et al., 2011; Wagner et al., 2020).

The transition analysis further supports the notion that optimal functioning declined for some participants over time. Although most individuals remained stable in their cluster membership, a greater number shifted from High to Low functioning than vice versa. This asymmetry suggests that the period between assessments — marked by the broader impact of the pandemic — may have influenced well-being trajectories, even in the absence of formal restrictions. These findings underscore the importance of longitudinal designs not only to predict outcomes, but also to monitor intra-individual changes in functioning over time.

### Limitations and Future Directions

With respect to the limitations of the study, three aspects could have influenced the presented results. First, self-report measures were used to assess the predictor and criterion variables, which may affect the validity of the data. In particular, academic achievement — one of the study’s key outcome variables — was also self-reported. Although participants were asked to report the official grades recorded in their transcripts, which they could easily access online, this method still relies on participant accuracy and honesty. The use of self-reported academic performance may introduce recall bias or social desirability effects, potentially affecting the reliability of this measure. For future studies, it would be advisable to obtain academic data directly from institutional records or to validate self-reported grades through cross-verification procedures.

Second, although the data were collected before the immediate period preceding the COVID-19 pandemic, the follow-up data were collected afterward. While there were virtually no COVID-19-related restrictions at the time of follow-up, this situation might have influenced participants' responses, as previously mentioned. It is important to note, however, that both assessments were conducted during periods without active restrictions, which provides a relatively equivalent context for the administration of the instruments and helps reduce potential biases due to differing external conditions. Third, the study was conducted with participants residing in the metropolitan region and attending a private university, predominantly women. While the results largely align with those of previous studies, with the exception of academic achievement, these characteristics may have affected the results and are recognized as limitations of the study’s conclusions. Additionally, the relatively homogeneous and elevated age of the participants — who were psychology students in the later years of a five-year degree program — reflects common patterns in Argentinian higher education, where it is frequent for students to begin or resume their university studies later in life. This demographic profile may limit the generalizability of the findings to traditional, younger undergraduate populations or to other academic disciplines. Fourth, although the analytical strategy employed — cluster analysis followed by logistic regression — was suitable for the aims and sample size of the study, it does not allow for modeling latent variables or accounting for measurement error, as would be possible with more complex approaches such as structural equation modeling (SEM). Future studies with larger and more heterogeneous samples could benefit from using SEM or longitudinal modeling techniques to test more nuanced relationships among variables over time.

Fourth, the lack of significant differences in academic achievement between clusters stands in contrast to previous findings that identified high factors — particularly tenacity and erudition — as relevant predictors of academic performance. While the homogeneity of the sample may partially explain this result, other methodological factors should also be considered. For instance, the study design prioritized participant retention across two time points over maximizing variability in academic performance, which may have reduced the ability to detect between-group differences. Additionally, unmeasured mediating variables — such as academic motivation, emotional regulation, or stress — may play a key role in the relationship between personality traits and academic outcomes. Future research should consider including these types of variables and aim for more diverse samples across academic disciplines, institution types, and performance profiles to better understand the role of high factors in predicting academic success.

Future studies should consider conducting longitudinal research with larger, more diverse samples and expanded methodological designs to examine whether these effects persist over time, under what conditions, and through which underlying mechanisms.

## Supplementary Materials

**Table:** 

Type of supplementary material	Availability/Access
Data	
Data Files	Castro-Solano and Garcia Dilba (2026)
Code	
R and SPSS codes	Castro-Solano and Garcia Dilba (2026)
Material	
Test Materials	Castro-Solano and Garcia Dilba (2026)
Study/Analysis preregistration	
Study was not preregistered	—
Other	
Codebook - Data dictionary	Castro-Solano and Garcia Dilba (2026)

## Data Availability

All the data, materials, analyses and codes used in this study are publicly available in the Open Science Framework (OSF) repository at Castro-Solano and Garcia Dilba (2026). This includes the complete dataset, R code/SPSS syntax for data preparation and analyses, a data dictionary linking each item to its full wording, a table with item translations into English, and all the study materials.

## Related Versions

This work is part of the doctoral thesis of the first author, titled *High factors of the High Five Model: Predicting psychological, academic, and occupational adjustment of university students*. Universidad de Palermo, Buenos Aires. Argentina.
